# Resistance to MPTP-Neurotoxicity in α-Synuclein Knockout Mice Is Complemented by Human α-Synuclein and Associated with Increased β-Synuclein and Akt Activation

**DOI:** 10.1371/journal.pone.0016706

**Published:** 2011-01-31

**Authors:** Bobby Thomas, Allen S. Mandir, Neva West, Ying Liu, Shaida A. Andrabi, Wanda Stirling, Valina L. Dawson, Ted M. Dawson, Michael K. Lee

**Affiliations:** 1 Neuroregeneration and Stem Cell Programs, Johns Hopkins University School of Medicine, Baltimore, Maryland, United States of America; 2 Department of Neurology, Johns Hopkins University School of Medicine, Baltimore, Maryland, United States of America; 3 Department of Pathology, Johns Hopkins University School of Medicine, Baltimore, Maryland, United States of America; 4 Department of Neuroscience, Johns Hopkins University School of Medicine, Baltimore, Maryland, United States of America; 5 Department of Physiology, Johns Hopkins University School of Medicine, Baltimore, Maryland, United States of America; Brigham and Women's Hospital, Harvard Medical School, United States of America

## Abstract

Genetic and biochemical abnormalities of α-synuclein are associated with the pathogenesis of Parkinson's disease. In the present study we investigated the *in vivo* interaction of mouse and human α-synuclein with the potent parkinsonian neurotoxin, MPTP. We find that while lack of mouse α-synuclein in mice is associated with reduced vulnerability to MPTP, increased levels of human α-synuclein expression is not associated with obvious changes in the vulnerability of dopaminergic neurons to MPTP. However, expressing human α-synuclein variants (human wild type or A53T) in the α-synuclein null mice completely restores the vulnerability of nigral dopaminergic neurons to MPTP. These results indicate that human α-synuclein can functionally replace mouse α-synuclein in regard to vulnerability of dopaminergic neurons to MPTP-toxicity. Significantly, α-synuclein null mice and wild type mice were equally sensitive to neurodegeneration induced by 2′NH_2_-MPTP, a MPTP analog that is selective for serotoninergic and noradrenergic neurons. These results suggest that effects of α-synuclein on MPTP like compounds are selective for nigral dopaminergic neurons. Immunoblot analysis of β-synuclein and *Akt* levels in the mice reveals selective increases in β-synuclein and phosphorylated *Akt* levels in ventral midbrain, but not in other brain regions, of α-synuclein null mice, implicating the α-synuclein-level dependent regulation of β-synuclein expression in modulation of MPTP-toxicity by α-synuclein. Together these findings provide new mechanistic insights on the role α-synuclein in modulating neurodegenerative phenotypes by regulation of *Akt*-mediated cell survival signaling *in vivo*.

## Introduction

Parkinson's disease (PD) is predominantly an idiopathic disorder without cure and with limited symptomatic treatment. PD is marked by a selective and progressive loss of dopaminergic neurons in the substantia nigra pars compacta (SNpc) characterized by movement abnormalities including resting tremor, bradykinesia, postural instabiltity and rigidity[1]. Currently, both environmental and genetic factors are implicated in the pathogenesis of PD [2]. The genetic cause for PD was first established with identification of mutations in the gene encoding for the synaptic protein α-synuclein in several families with the autosomal dominant form of PD [3], [4], [5], [6]. A relationship between α-synuclein and PD is further suggested by the finding that α-synuclein is the main structural component of cytoplasmic proteinaceous inclusion bodies (Lewy bodies) and neurites (Lewy neurites) that are characteristic of both sporadic and familial PD cases [7]. Further, transgenic drosophila overexpressing human α-synuclein demonstrates selective dopaminergic neuronal dysfunction [8] and forced overexpression of human α-synuclein in rodents show varying degrees of neuronal dysfunction and degeneration [9], [10], [11], [12], [13] including dopaminergic degeneration [14], [15], [16], [17], [18]. However, a variety of human α-synuclein transgenic mice with increased α-synuclein aggregation, motoric dysfunction, and neurodegeneration have failed to show overt neurodegeneration of dopaminergic neurons [10], [11], [12]. Thus, the mechanistic basis for α-synuclein abnormalities and dopaminergic degeneration is unresolved.

In addition, the relationship of α-synuclein abnormalities to other *in vivo* models of PD remains controversial. A widely used animal model of parkinsonism utilizes the selective dopaminergic neurotoxin MPTP that replicates the selective neuronal loss seen in PD [19], [20], [21]. A potential role for α-synuclein abnormalities in the pathogenesis of MPTP toxicity is suggested from the findings that α-synuclein aggregation occurs in mouse and primate models of MPTP-induced parkinsonism [21], [22], [23]. However, studies using rodents with increased expression of human α-synuclein have led to conflicting results where some studies show lack of increased sensitivity to MPTP with human α-synuclein expression [24], [25] and some studies showing increased sensitivity/abnormality associated MPTP in human α-synuclein transgenic mice [26], [27]. The discordance between these studies may be associated with number of factors including differences in MPTP toxicity paradigm used, promoters used, mouse strains, and the age of animals used for the MPTP treatment. Lack of more robust and consistent increase in MPTP toxicity with the overexpression of human α-synuclein is puzzling since several studies show that the expression of endogenous mouse α-synuclein is required to sensitize dopaminergic neurons to MPTP [21], [28], [29], [30], [31]. One of the possibilities is that human α-synuclein does not function in the MPTP toxicity pathway in rodents.

In this report, we have attempted to more fully document the *in vivo* relationship between α-synuclein expression and vulnerability of dopaminergic neurons to MPTP. We used multiple transgenic lines at multiple conditions to confirm that increased expression of human α-synuclein is not associated with robust increase in the susceptibility of the human α-synuclein transgenic mice to MPTP toxicity. However, human α-synuclein is able to restore the MPTP-sensitivity of the dopaminergic neurons lacking mouse α-synuclein expression, showing that human α-synuclein can functionally replace mouse α-synuclein in regard to vulnerability of dopaminergic neurons to MPTP-toxicity. Significantly, the mouse α-synuclein null mice are not protected from neurodegeneration induced by 2′NH_2_-MPTP, the MPTP analog with selective toxicity to central noradrenergic and serotoninergic neurons [32], [33]. This indicates that the MPTP resistance phenotype of mouse α-synuclein null mice is selective for dopaminergic neurons and that alterations in the general metabolism and/or trafficking of MPP+ in the synaptic terminal are not involved in MPTP resistant phenotype of the mouse α-synuclein null mice. We also find that the levels of β-synuclein and phosphorylated *Akt* is inversely correlated with α-synuclein expression in the nigro-striatal system and increased β-synuclein and phosphorylated *Akt* (*pAkt*) levels correlate with the protection from MPTP toxicity. Thus, we propose that regulation of β-synuclein and *Akt* levels by α-synuclein expression is an important contributor to regulation of MPTP-toxicity by α-synuclein.

## Results

### Increased expression of human α-synuclein does not increase vulnerability of dopaminergic neurons to MPTP-intoxication

#### Acute MPTP paradigm

**Figure 1:**
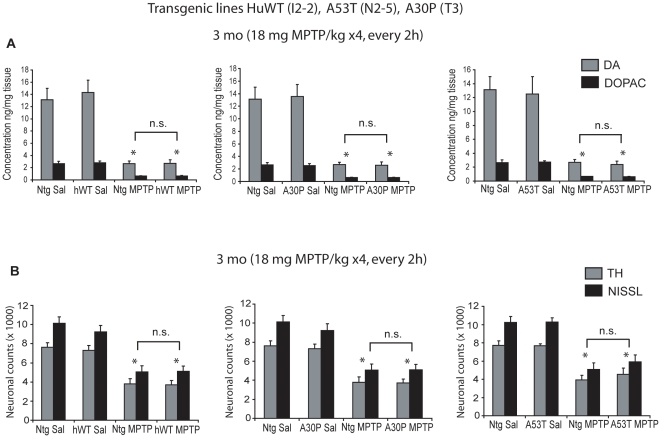
Nigrostriatal system in transgenic wild type and mutant human α-synuclein overexpressing mice do not show increased sensitivity to acute MPTP intoxication. Transgenic mice expressing moderate levels (∼3-fold over endogenous levels) of wild type (WT, line I2-2), A30P mutant (line T3), and A53T mutant (line N2-5) human α-synuclein were subjected to acute paradigm of MPTP (18 mg MPTP/kg free base X4, every 2h). *A.* Striatal levels of dopamine and major metabolite DOPAC in transgenic and non-transgenic littermate cohorts were analyzed 7 days after the last MPTP injection. No differences were seen among transgenic and non-transgenic mice receiving the same treatments (saline or MPTP). *B.* Stereologic neuronal counts of TH-immunopositive and total neurons in transgenic and non-transgenic littermate cohorts analyzed two weeks following the last MPTP injection. While MPTP treatments caused a significant reduction in TH-immunopositive and total neurons, no significant differences in neuronal counts are observed between transgenic and non transgenic mice receiving the same treatment (saline or MPTP). Data represent mean ± SEM. *p<0.05, statistical significance versus saline controls using two way ANOVA, n = 5-6 per group, n.s not significant.

Eleven to thirteen week old transgenic mice generated using the mouse prion promoter, overexpressing wild type (line I2-2) or mutant A53T (line N2-5) or mutant A30P (line T3) human α-synuclein, and non transgenic littermates received either an acute paradigm of MPTP (18 mg MPTP/kg free base X4, every two hours) or saline. One week following treatment, HPLC with electrochemical detection demonstrates a profound reduction in total striatal dopamine following an acute paradigm of MPTP-treated mice compared to saline controls (**Fig. 1A**) (ANOVA, p<0.05). Analysis of striatal levels of dopamine and its metabolite DOPAC showed no differences in the losses between any of the transgenic lines and non-transgenic cohorts following MPTP administration. MPTP treatment also results in increased striatal dopamine turnover (DOPAC/DA) which is not statistically different in any of the transgenic lines compared to non-transgenic animals (data not shown). Stereologic counts of total neurons (Nissl positive) and TH-immunopositive neurons in substantia nigra show that MPTP treatment results in a significant decrease in total and TH-immunopositive neurons of the SNpc in all lines of mice (**Fig. 1B**) (ANOVA, p<0.05). However, no differences in susceptibility to MPTP are observed between transgenic versus non-transgenic mice.

#### Sub- acute MPTP paradigm

**Figure 2:**
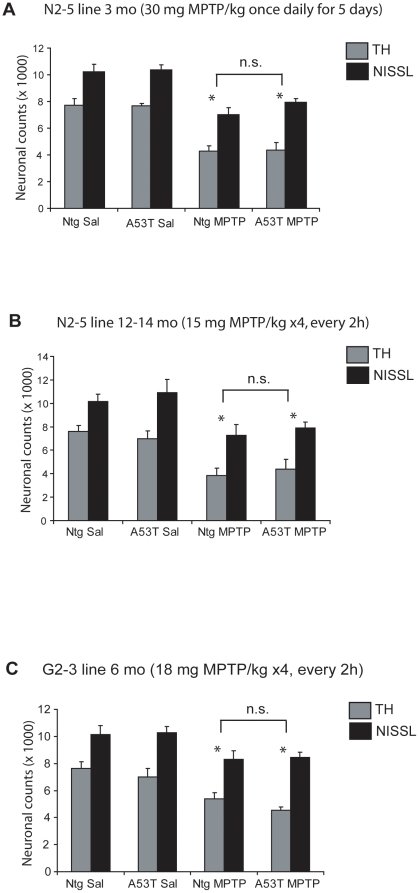
Effects of sub acute MPTP regimen, aging, and transgene expression level on the MPTP sensitivity of nigral dopaminergic neurons in human A53T α-synuclein transgenic mice. *A.* Stereologic cell counts of total and TH-immunopositive neurons of SNpc in non transgenic and human A53T transgenic mice (line N2-5, ∼3-fold) analyzed two weeks after a sub-acute paradigm of MPTP (30 mg MPTP/kg free base once a day for 5 days). Although sub acute MPTP caused significant reductions in total and TH-positive neurons no significant differences were seen among transgenic and non transgenic mice receiving the same treatments (saline or MPTP). *B*. TH-positive and total neuronal counts in SNpc of 12-14 month old non transgenic and human A53T transgenic mice (line N2-5) after acute MPTP (15 mg MPTP/kg free base X4, every 2 h) at 7 days. Acute MPTP in older mice caused significant reduction in total and TH-positive neurons no significant differences were seen among transgenic and non transgenic mice receiving the same treatments (saline or MPTP). *C*. Cell counts of TH-positive and total neurons in high expressing lines of human A53T transgenic mice (G2-3 line, ∼6-fold) 7 days after acute MPTP intoxication (18 mg MPTP/kg free base X4, every 2 h). MPTP-intoxication resulted in a significant reduction of total and TH-positive neuronal counts in non transgenic and high expressing lines of A53T transgenic mice compared to saline treatments. A moderate increase in the vulnerability was observed in A53T transgenic mice. Data represent mean ± SEM. *p<0.05, statistical significance versus saline controls using two way ANOVA, n = 5-6 per group, n.s., not significant. #, significant using Neuman-Keuls post-test (*p<0.05*) but not with the Tukey-Kramer post test.

It is suggested that the paradigm of MPTP delivery may have an effect upon mechanisms of neuronal death. Sub-acute MPTP paradigms may cause apoptotic mechanisms of neuronal death [34], [35], [36] not seen in acute paradigms, which causes necrotic cell death [37]. Thus, we treated eleven to thirteen weeks old transgenic mice, expressing the A53T mutant human α-synuclein (line N2-5), and non-transgenic litter mates with a 5 day sub-acute MPTP paradigm (30 mg MPTP/kg free base once daily for five days). Stereologic neuronal counts were performed 2 weeks following the final MPTP dose (**Fig. 2A**). Although, a significant loss of total and TH-immunopositive neurons were seen following MPTP administration compared to saline controls (ANOVA, p<0.05), no difference was seen among transgenic and non-transgenic lines. Identical results were obtained with the A30P mutant human α-synuclein transgenic mice treated with the sub-acute MPTP paradigm (data not shown).

#### Effect of age on the susceptibility of dopaminergic neurons to MPTP

Because aging is associated with increased vulnerability to MPTP-intoxication [38], [39] and increased vulnerability to α-synuclein dependent neurodegeneration [11], we examined if age had a synergistic effect with over expression of mutant human α-synuclein on the susceptibility to nigral dopaminergic neurons following MPTP-intoxication. Transgenic and non-transgenic mice from the N2-5(A53T) line were treated with an acute MPTP paradigm (15 mg MPTP/kg free base X 4, every 2 hours) at 12–14 months of age. A lower than usual dose of MPTP was used because of increased sensitivity of aged mice to MPTP. Again, despite the significant loss of SNpc neurons in MPTP treated mice (ANOVA, p<0.05), no increase in susceptibility is observed in transgenic overexpressors versus non-transgenic cohorts (**Fig. 2B**).

#### Effect of high expression levels of A53T in mice on the susceptibility of dopaminergic neurons to MPTP

To be certain that overexpression of human α-synuclein does not affect MPTP susceptibility, we also examined the MPTP susceptibility of the A53T transgenic mice expressing very high levels of A53T human α-synuclein. Transgenic mice from line G2-3(A53T) express the transgene at approximately six times the level of endogenous mouse α-synuclein and develop a fatal neurodegenerative disease with an average life span of ∼12 months [11]. To investigate if this higher level of expression had a synergistic effect with MPTP on neuronal toxicity, 6 month old transgenic and non-transgenic littermates were treated with acute MPTP paradigm (18 mg MPTP/kg free base X 4, every 2 hours) and numbers of neurons in SNpc were determined (**Fig. 2C**). Analysis of cell counts show that MPTP treatment of A53T transgenic mice (65%) may result in a slightly lower number of dopaminergic neurons than with the non-transgenic littermates (70.4%) (**Fig. 2C**). Specifically, the difference in number of TH-immunopositive neurons between the MPTP treated non-transgenic mice and A53T transgenic mice are significant when the ANOVA analysis performed with the Neuman-Keuls-post-test (p<0.05). However, the differences are not significant when ANOVA analysis with Tukey-Kramer posthoc test was applied. Collectively, these results indicate that the increased expression of human α-synuclein (either wild type or mutant) do not significantly affect MPTP susceptibility and, at best, very high levels of A53T mutant human α-synuclein is required to slightly increase the vulnerability of dopaminergic neurons to MPTP-toxicity.

### Human α-synuclein complements MPTP-resistance phenotype but not the developmental reduction of dopaminergic neurons in α-synuclein null mice

The lack of increased MPTP susceptibility with the human α-synuclein transgenic mice is surprising since decreased expression of mouse α-synuclein is associated with significant protection from MPTP-intoxication [21], [29], [30], [31], [40]. One possibility is that human and mouse α-synuclein function differently regarding MPTP-susceptibility in mice.

**Figure 3:**
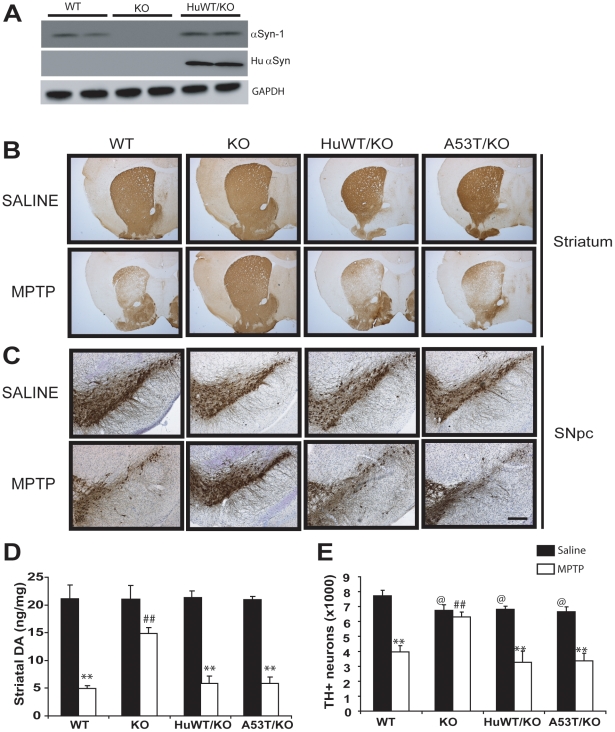
Human α-synuclein complements MPTP resistant phenotype of mouse α-synuclein null mice. ***A.*** Expression of mouse and human α-synuclein in wild type (WT), mouse α-synuclein nulls (KO) and mice expressing wild type human α-synuclein (line I2-2) on a mouse α-synuclein null background (hWT/KO), total proteins isolated from 9 month old striata were subjected to immunoblot analysis for total α-synuclein (Syn-1), human α-synuclein (HuSyn), and GAPDH. ***B and C.*** TH-immunostaining staining of striatum (B) and SNpc (C) 7 days following saline or acute MPTP (20 mg/kg free base four times every 2 hour) treatment in wild type (WT), mouse α-Syn nulls (KO) and human α-Syn transgenic [wild type (hWT, line I2-2) and A53T (line N2-5)] on the mouse α-Syn null background (hWT/KO; A53T/KO). Only the KO is protected from MPTP toxicity. ***D and E***. MPTP intoxication results in significant reductions in striatal DA-levels ***(D)*** and TH-positive neuronal cells ***(E)*** in mice with either mouse or human α-Syn expression. The α-Syn KO mice were completely protected against MPTP. The reduced basal number of DAergic neurons in α-Syn KO mice was not complemented by the human α-Syn expression ***(E)***. Data represent mean ± SEM. ^@,^** *p<0.05*, versus saline and ^#^ *p<0.05*, versus wild type MPTP, Two way ANOVA, n = 5-6, n.s not significant, scale bar: 200 µm.

To test the above possibility, we examined whether expression of human α-synuclein could restore the susceptibility of dopaminergic neurons to MPTP toxicity in mouse α-synuclein null mice. To test whether human α-synuclein expression can complement the MPTP-resistant phenotype of the mouse α-synuclein null mice, human α-synuclein transgenic mice (WT and A53T) were successively crossed to the mouse α-synuclein null mice [41] to generate human α-synuclein transgenic mice lacking mouse α-synuclein expression (**Fig. 3A**). The resulting mice were subjected to acute MPTP intoxication and nigrostriatal dopaminergic neurotoxicity was studied by performing stereologic neuronal counts of SNpc and quantitation of striatal dopamine and its metabolites 1 week after MPTP (20 mg MPTP/kg free base X4, every 2 hours) in 6–8 week old male mice. Consistent with previous reports [21], [28], [30], the α-synuclein null mice are protected against MPTP-induced neurodegeneration at both striatum and SNpc **(** **Fig. 3B, C**). MPTP-induced reductions in striatal metabolites of dopamine, DOPAC and HVA and also increased dopamine turnover (DOPAC+HVA/DA) are blocked in α-synuclein null mice (data not shown). However, mice expressing human α-synuclein (wild type and A53T) on the mouse α-synuclein null background show significant sensitivity to MPTP **(** **Fig. 3B-E** **)**. The loss of striatal dopamine and nigral dopaminergic neurons following MPTP treatment in human α-synuclein/mouse α-synuclein null mice were comparable to that seen with the wild type animals (**Fig. 3D,E**). Furthermore, human α-synuclein/mouse α-synuclein null mice were equally sensitive as the wild type mice on the levels of dopamine metabolites following MPTP (data not shown).

The finding that MPTP-neurotoxicity resistant phenotype of the mouse α-synuclein null mice is completely reversed by the expression of human α-synuclein transgenes show that the lack of robust enhancement in MPTP neurotoxicity in human α-synuclein transgenic mice is not because human α-synuclein can not function in the MPTP toxicity pathway. A likely possibility is that the level of mouse α-synuclein is saturating and the additional α-synuclein expression does not significantly increase the effects of α-synuclein on susceptibility to MPTP neurotoxicity.

Significantly, while the human α-synuclein can complement mouse α-synuclein in regard to MPTP neurotoxicity, human α-synuclein does not completely complement all of the phenotypes of mouse α-synuclein null mice. Mouse α-synuclein null mice have significantly fewer TH+ neurons in SNpc (**Fig. 3E**) due to developmental reduction in the number of TH+ neurons generated [31] Expression of human α-synuclein in mouse α-synuclein null mice did not restore the number of TH+ neurons to the level of non-transgenic mice (**Fig. 3E**).

### Decreased vulnerability of α-synuclein null mice to intoxication is selective for dopaminergic neurons

It's believed that α-synuclein null mice are protected against MPTP intoxication because lack of α-synuclein expression affects a yet to be defined process that occurs post uptake of MPP+ into the terminal and pre-mitochondrial complex I inhibition [42]. Based on this hypothesis, potential factors that can be affected by the loss of α-synuclein expression and also modulate MPTP toxicity is the activity of the vesicular monoamine transporter 2 (VMAT2) and the ability of MPP+ to access mitochondrial complex-I. To test whether these factors could be involved, we examined whether the serotoninergic and noradrenergic neurons in mouse α-synuclein null mice were resistant to 2′NH_2_-MPTP neurotoxicity.

**Figure 4:**
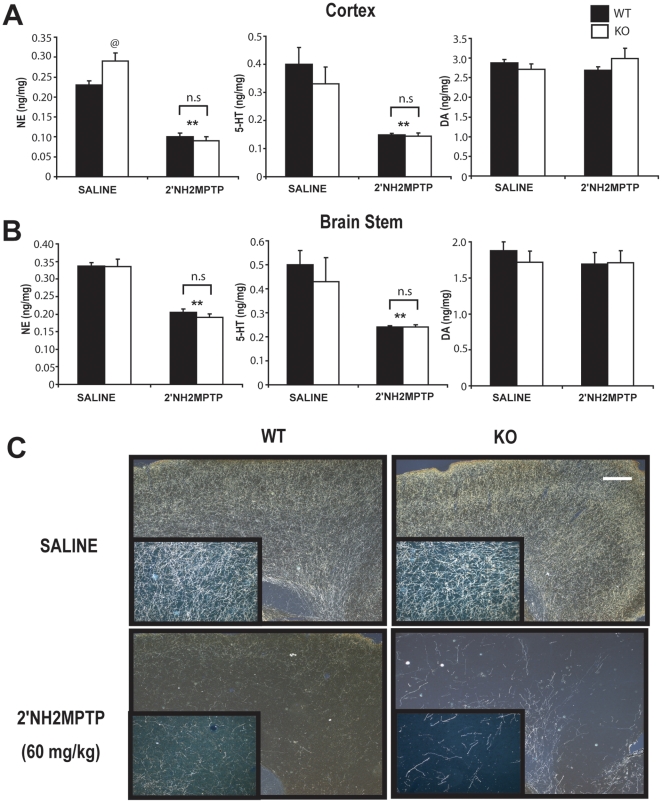
α-synuclein null mice are not protected against noradrenergic and serotoninergic neurotoxin 2′NH_2_-MPTP. ***A and B.*** Cortical ***(A)*** and brainstem ***(B)*** levels of norepinephrine (NE), serotonin (5-HT) and dopamine (DA) were determined two weeks after saline or acute 2′NH2-MPTP (15 mg/kg X4, every 2 hours) treatment in wild type (WT) and α-synuclein null (KO) mice. 2′NH2-MPTP treatment lead to significant loss of NE and 5-HT but not DA in cortex and brain stem. ***C.*** 2′NH2-MPTP treatment resulted in a significant loss of cortical serotonergic fibers both in wild type and α-synuclein null mice. Unlike MPTP, the α-synuclein null mice are not resistant to the neurotoxic insult of serotonergic and nor adrenergic neurotoxin 2′NH2-MPTP. Data represent mean ± SEM. ** p<0.05, statistical significance versus saline controls using two way ANOVA, n = 6-7 per group, n.s., not significant. Scale bar: 100 µm.

Intoxication with 2′NH_2_-MPTP leads to degeneration of serotoninergic and noradrenergic neurons without affecting dopaminergic neurons (**Table 1** **and** **Fig. 4A and B**) [32]. The mode of 2′NH_2_-MPTP toxicity is virtually identical to that of MPTP [33], [43], [44]. Thus, we reasoned that if α-synuclein modulates a general synaptic trafficking of MPP+, mouse α-synuclein null mice should be resistant to 2′NH_2_-MPTP toxicity. Mouse α-synuclein null and the littermate wild type mice were generated by mating two mice that are both heterozygous for the mouse α-synuclein gene. Six to eight week old male mice were treated with 2′NH_2_-MPTP (15 mg 2′NH_2_-MPTP/kg X4, every 2 hours) and the levels of serotonin and norepinephrine in the cortex, striatum, and brain stem were determined 14 days following the last 2′NH_2_-MPTP treatment. Our results show that 2′NH_2_-MPTP treatment leads to the profound reductions in the serotonin and norepinephrine levels in all brain regions. The magnitudes of reductions in neurotransmitter levels were comparable between wild type and α-synuclein null mice **(** **Fig. 4A, B** **)**. Analysis of serotoninergic innervations by immunohistochemical analysis confirms the significant loss of cortical serotoninergic fibers following 2′NH_2_-MPTP treatments in both groups of mice **(** **Fig. 4C** **)**. Qualitatively, the losses of serotoninergic fibers were notably greater in the α-synuclein null mice than in the wild type controls.

**Table 1:** Levels of striatal dopamine and its metabolites in wild type and α-synuclein knock out mice following 2′NH_2_MPTP treatment.

Treatmentgroups	DA[ng/mg]	DOPAC[ng/mg]	HVA[ng/mg]	DOPAC+HVADA
**Saline** **[WT]**	20.3±2.5	1.90±0.06	1.74±0.04	0.18±0.01
**Saline** **[KO]**	19.4±1.5	1.87±0.05	1.68±0.05	0.18±0.01
**2′NH_2_MPTP [WT]**	21.2±3.5	2.1±0.09	1.6±0.05	0.17±0.02
**2′NH_2_MPTP [KO]**	20.5±4.5	1.9±0.08	1.73±0.08	0.17±0.03

Striatal levels of dopamine and its metabolites measured after 2 weeks of acute 2′NH_2_MPTP (15 mg/kg, i.p.) administered four times every two hours in wild type and α-synuclein knock out mice. Data are expressed as mean (n = 5) ± SEM. Statistical analysis was performed by ANOVA, revealing no significant differences among group means. DA, dopamine; DOPAC, 3,4-dihydroxy-phenylacetic acid; HVA, homovanillic acid, WT, wild type; KO, knock out.

The lack of resistance to the 2′NH_2_-MPTP toxicity in the α-synuclein null mice suggests that MPTP resistance phenotype of mouse α-synuclein null mice is selective for dopaminergic neurons. Further, our results suggest that alterations in the general metabolism and/or trafficking of MPP+ in the synaptic terminal are not involved in MPTP resistant phenotype of the mouse α-synuclein null mice. Even if such a phenomenon does exist, it might be due to yet undefined inherent mechanisms that differ in dopaminergic and serotoninergic/noradrenergic neurons that affect the general metabolism or trafficking of MPP+.

### The levels of β-synuclein and phosphorylated *Akt* are regulated by α-synuclein expression in ventral midbrain

Previously, increased expression of β-synuclein was found in the mouse α-synuclein null and mouse γ-synuclein null mice [31]. Because, β-synuclein has been shown to protect neurons from cell death, potentially via regulation of *p53* [45], by reducing α-synuclein protein expression [46], [47] and *Akt*-phosphorylation [48], [49], increased levels of β-synuclein in α-synuclein null mice could be a potential factor involved in the protection of dopaminergic neurons from MPTP toxicity.

**Figure 5:**
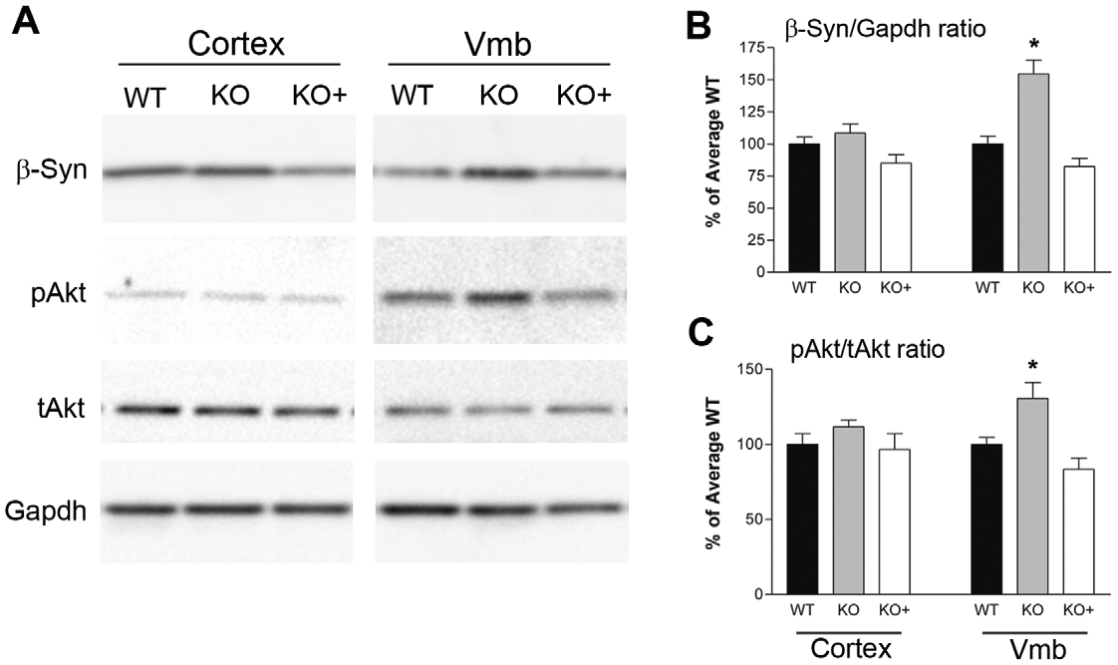
Levels of α-synuclein affects β-synuclein expression and basal *Akt* phosphorylation *in vivo*. ***A.*** Total tissue extracts from cortex and ventral midbrain of wild type (WT), moSyn-null mice (KO), and HuSyn transgenic (line I2-2) on moSyn-KO background (KO+) were immunobloted for endogenous β-synuclein, *Akt* (pSer473 and total) and GAPDH. ***B and C.*** Semi-quantitative analysis of β-synuclein **(B)** and pSer473-*Akt* levels ***(C)***. The values are mean ± SEM from 4 animals (*p>0.05, **p>0.01, ANOVA with Newman-Keuls post-test).

To further explore this hypothesis, we examined whether the increased levels of β-synuclein is regionally selective and whether the increase in β-synuclein levels in α-synuclein null mice could be reversed by expression of human α-synuclein. To determine whether β-synuclein levels are increased in brains of α-synuclein null mice, SDS-soluble proteins from cortex and ventral midbrain were subjected to immunoblotting for β-synuclein **(** **Fig. 5A** **)**. Semi-quantitative analysis show that, relative to the β-synuclein levels in wild type mice, the ventral midbrain, but not cortex, shows increased levels of β-synuclein in α-synuclein null mice **(** **Fig. 5B** **)**. Significantly, the level of β-synuclein is returned to the wild type levels by expressing human α-synuclein in the α-synuclein null background. Additionally, analysis of SDS soluble proteins in other brain regions like dorsal medial brain stem did not significantly affect β-synuclein levels for all the genotypes in comparison to wild type mice (data not shown).

Previous studies [47], [48], [49] showed that β-synuclein can provide neuroprotection via direct interaction with *Akt* and enhancing the accumulation of phosphorylated *Akt*. Thus, we examined whether the levels of p*Akt*(Ser473) was altered as a function of α-synuclein expression in the lines of transgenic mice. Immunoblot analysis for p*Akt*(Ser473) and total *Akt* shows that the level of p*Akt*(Ser473) is increased in the ventral midbrain but not in cortex **(** **Fig. 5A** **)**. As with β-synuclein expression, transgenic expression of human α-synuclein on the α-synuclein null background lead to decrease in p*Akt*(Ser473) levels **(** **Fig 5B** **)**.

Collectively, our results support the hypothesis that the increased expression of β-synuclein in α-synuclein null mice lead to increased level of p*Akt*(Ser473), contributing to neuroprotection in α-synuclein null mice. The finding that β-synuclein and p*Akt*(Ser473) levels are selectively increased only in the ventral midbrain of the α-synuclein null mice support our hypothesis that dopaminergic neurons are selectively protected from MPTP-like toxins in α-synuclein null mice.

## Discussion

Both α-synuclein and MPTP are believed to cause dopaminergic abnormalities that are relevant to the pathogenesis of PD, and hence we explored the relationship between α-synuclein expression and vulnerability of dopaminergic neurons to MPTP neurotoxicity. We show that the increased expression of human α-synuclein is not associated with increased vulnerability of dopaminergic neurons to MPTP toxicity. The lack of effect is not because human α-synuclein does not function in the MPTP toxicity pathway since human α-synuclein is able to completely restore the MPTP-resistant phenotype of the mouse α-synuclein null mice [21], [28], [29], [30], [31]. However, human α-synuclein does not completely replace mouse α-synuclein since the developmental reduction in the dopaminergic neurons is not complemented by human α-synuclein. One plausible explanation to this could be that the human α-synuclein might not be expressed in all cells at similar levels and times as the endogeneous mouse α-synuclein gene. However, we show that the lack of α-synuclein expression is not associated with protection from the 2′NH_2_-MPTP toxicity, indicating that the dopaminergic neurons are selectively protected from toxin-induced neurodegeneration in the mouse α-synuclein null mice. Finally, α-synuclein level dependent regulation of β-synuclein and p*Akt*(Ser473) expression in ventral midbrain, but not in cortex, suggest that MPTP-resistant phenotype of α-synuclein null mice involves neuroprotection by β-synuclein/p*Akt*.

Previous examinations of human α-synuclein expression and the vulnerability to MPTP have been variable. In order to control some of the sources of potential variation, we examined the MPTP-sensitivity of dopaminergic neurons in human α-synuclein transgenic mice as functions of MPTP neurotoxicity paradigms (acute and sub-acute), human α-synuclein variants, human α-synuclein levels, and age. Using two standard paradigms (acute and sub acute) of MPTP neurotoxicity that results in significant loss of dopaminergic neurons in non-transgenic controls, we observe that wild type and mutant human α-synuclein transgenic mice demonstrate comparable sensitivity to MPTP. However, very high levels of A53T mutant human α-synuclein in transgenic mice may be associated with a modest increase in the MPTP-dependent neurodegeneration. Overall, our results are consistent with the other reports that fail to demonstrate a synergistic effect of human α-synuclein with MPTP [24], [25]. However, one study showed that A30P transgenic mice are more sensitive to MPTP neurotoxicity but not to rotenone induced dopaminergic neurodegeneration [27]. A significantly attenuated neurotoxicity in nontransgenic mice (i.e., very little loss of dopaminergic neurons with the dose of MPTP causing ∼50% reduction in our study) observed in this study suggest that there may be significant strain background and/or other transgene specific effects [27]. Overall, the general lack of increase in the vulnerability of dopaminergic neurons as a function of increased expression of human α-synuclein suggest that even at high levels of expression, human α-synuclein does not cause generalized stress to dopaminergic neurons that is sufficient to affect the MPTP toxicity paradigm used in this study. However, we can not exclude the possibility that increased expression of human α-synuclein would sensitize the dopaminergic neurons to more chronic toxicity paradigms. A major difference between MPTP-induced parkinsonism and idiopathic PD is time of progression for symptom development, thus it is possible for acute nature of MPTP toxicity paradigm used in this and other studies precludes pathological interaction between α-synuclein and MPTP toxicity [24], [25]. This notion is supported by the results showing that the expression of mutant α -synuclein is associated with the increased vulnerability of dopaminergic neurons to chronic application of manganese/paraquat [50].

Although, human α-synuclein and MPTP both can cause neurodegeneration, it is plausible that these effects do not directly interact. Arguments against this line of reasoning, however, include findings of increased α-synuclein levels in mice SNpc following MPTP, increased nitration of α-synuclein in mouse SNpc following MPTP, increased α-synuclein aggregates in SNpc of baboons following MPTP and *in vitro* studies of α-synuclein toxicity [22], [51], [52], [53]. In particular, a number of *in vitro* studies show that human α-synuclein overexpression was associated with enhanced cell death following exposure to MPP^+^, the toxic metabolite of MPTP [54], [55]. Finally, studies have shown gene dose dependent reductions in the susceptibility to MPTP in α-synuclein null mice [21], [28], [29], [30], [31]. Given these findings, the expectation was that increased levels of α-synuclein would potentiate MPTP neurotoxicity. The lack of enhanced vulnerability to MPTP neurotoxicity with human α-synuclein transgenic mice raised the possibility that human α-synuclein and mouse α-synuclein do not function equally in sensitizing dopaminergic neurons to MPTP toxicity *in vivo*. However, our results show that human α-synuclein can functionally replace mouse α-synuclein and complements the MPTP resistant phenotype of the mouse α-synuclein null mice. Thus, it appears that the endogenous levels of mouse α-synuclein expression is saturating in respect to sensitizing the dopaminergic neurons to MPTP toxicity.

Another significant finding in our study is that serotoninergic and noradrenergic neurons in the mouse α-synuclein null mice are exquisitely sensitive to 2′NH_2_-MPTP toxicity. Given that the 2′NH_2_-MPTP toxicity is mechanistically very similar to the MPTP toxicity, lack of resistance to 2′NH_2_-MPTP in the α-synuclein null mice indicate that the MPTP resistance of α-synuclein null mice is not because of alterations in the general synaptic metabolism/targeting of MPP+. This view is supported by the study showing that α-synuclein null mice are resistant to other mitochondrial toxins (malonate and 3-nitropropionic acid) that affect nigrostriatal dopaminergic neurons [56]. In this study, it was proposed that attenuated mitochondrial toxin induced release of dopamine in α-synuclein null mice [28] may be an important factor in neuroprotection from mitochondrial toxin. In addition to the potential effects of α-synuclein on dopamine release, our results suggest that the lack of α-synuclein leads to selective protection of nigrostriatal dopaminergic neurons from MPTP-like toxins. Because the expression of β-synuclein, which has been shown to be neuroprotective [45], [46], [48], [49], is increased in the ventral midbrain of α-synuclein null mice **(** **Fig. 5** **)**, it seems likely that β-synuclein expression and subsequent activation of *Akt* are important contributors to the MPTP-resistant phenotype of α-synuclein null and γ-synuclein null mice [31]. This view is consistent with the protection of dopamine neurons from 6-OHDA toxicity by expression of a constitutively active *Akt* mutant [57]. Moreover, α/β/γ-synuclein-dependent regulation of *Akt* may provide a mechanistic basis for modulation of dopaminergic neurotransmission by synuclein. Specifically, regulation of *Akt* signaling modulates dopamine-mediated locomotion in mice, including amphetamine-induced locomotion, and impaired *Akt* signaling is implicated in schizophrenia [58], [59]. Thus, increased activation of *Akt* in α-synulcein-null mice may account for the impaired amphetamine-induced locomotor response of α-synuclein-null mice [41]. Finally, since increased α-synulcein expression and decreased β-synuclein expression are associated with PD and other α-synucleinopathies [5], [60], regulation of *Akt* activity by synuclein expression may be an important factor in the pathogenesis of human α-synucleinopathies. Notably, other familial PD-linked gene products, parkin [61], DJ-1 [62], [63], and PINK1 [64], [65] are also associated with *Akt* signaling, supporting an intriguing notion that alterations in *Akt* signaling may be a common pathologic factor in PD.

In summary, our results are consistent with the notion that α-synuclein expression has significant cell biological consequences for dopaminergic neurons and some of these effects, such as vulnerability to MPTP-like toxins and regulation of β-synuclein expression, appear to be specific for dopaminergic neurons. In particular, given the important implications of our finding that α-synuclein expression regulates β-synuclein expression in nigrostriatal system, it will be important to further study cell biological basis for how α-synuclein regulates β-synuclein expression. Further, our studies provide a strong rationale for critical examination of the mechanistic relationship between *Akt* activation and MPTP toxicity. Finally, given that β-synuclein can attenuate α-synuclein toxicity *in vivo* [46], [47], [48], [49], it is attractive to propose that regulation of endogenous β-synuclein expression and *Akt* activation may be a significant factor in the transgenic animal models of α-synucleinopathy and in human α-synucleinopathies.

## Materials and Methods

### Animals

All mice were housed and treated in strict accordance with the National Institutes of Health *Guide for Care and use of Laboratory Animals*. Mice were housed in a pathogen free facility, about 4-5 animals per cage in a temperature controlled room with a 12 hour light/dark cycle and with food and water *ad libitum*. All procedures were approved by and conformed to the guidelines of the Institutional Animal Care Committee of Johns Hopkins University.

Transgenic lines of mice were generated using the MoPrP promoter as previously described to over express human α-synuclein (WT, line I2-2, ∼3-fold increase in total α-synuclein level), A53T mutant human α-synuclein (A53T, line N2-5 and G2-3, ∼3-fold and ∼6-fold increase, respectively) and A30P mutant human α-synuclein (A30P, line T3 and O2, ∼3-fold and 10-fold increase, respectively) [11]. Non transgenic littermate cohorts were also generated for each of these lines to account for potential genetic differences. The transgenic mice used were N3-N5 generation backcrossed to C57/BL6 strain.

Human α-synuclein transgenic mice lacking mouse α-synuclein expression was generated by successive mating of human α-synuclein transgenic mice, congenic for C57BL6 strain (N10), to mice lacking endogenous α-synuclein [41] that were procured from Jackson labs. To generate the cohorts for the MPTP treatment, mice that are double heterozygous for human α-synuclein and mouse α-synuclein were mated to mice heterozygous for mouse α-synuclein. The resulting progenies contained all of the possible genotypes. The resulting male non-transgenic (wild type), mouse α-synuclein null, and human α-synuclein transgenic on mouse α-synuclein null background littermates were used for the current study.

### MPTP and 2′NH_2_-MPTP treatment in mice

Male mice were used for neurotoxic challenges induced by dopaminergic neurotoxin MPTP and its structural analogue 2′NH_2_-MPTP, which is a selective neurotoxin for central serotoninergic and noradrenergic neuronal systems. Two different MPTP paradigms were used in the present study. For the acute paradigm, mice were administered four acute doses of MPTP (20 mg/kg, 18 mg/kg, and 15 mg/kg free base) every two hours four times a day and analyzed 7 days post-treatment. For sub-acute MPTP paradigm, mice were treated with 30 mg/kg free base MPTP daily for a total of 5 days and toxicity analyses performed two weeks after the last MPTP injection. To assess neurotoxicity to central serotoninergic and noradrenergic system mice were treated with 2′NH_2_-MPTP in an acute paradigm of 15 mg/kg 2′NH_2_-MPTP divided into four doses separated by 2 hours and the toxicity analyses were performed two weeks later. Control cohorts of mice received equivalent volumes of saline at the same frequency as the respective paradigms of MPTP or 2′NH_2_-MPTP used in the study. All systemic injections were administered through the intraperitoneal route and the neurotoxins were dissolved in normal saline. All procedures involving MPTP and 2′NH_2_-MPTP injections in mice were performed according to standard procedures [66].

### Immunohistochemistry

Immunohistochemical analyses for tyrosine hydroxylase expression was performed using methods previously described [67] using a rabbit anti-tyrosine hydroxylase antibody (Novus Biologicals, Littleton, CO). Serotoninergic axons were visualized by enhanced immunocytochemistry with an anti-serotonin antibody using an established method [68]. Briefly, formaldehyde fixed frozen sections were incubated with the rabbit anti-serotonin antibody (1∶12,500, Incstar, Stillwater, MN) followed by the secondary antibody [1∶1000 biotin-labeled F(ab')_2_ fragment goat-anti rabbit antibody (Jackson Immunoresearch, West Grove PA)] and visualized by incubation in biotin-streptavidin-HRP (Vector Laboratories, Burlingame, CA) and SigmaFast™ DAB Peroxidase Substrate (Sigma, St. Louis, MO). Sections were mounted on glass slides and dehydrated in graded ethanol, followed by chloroform: ethanol (1∶1) and rehydrated. The sections were then incubated in 1.5% sliver nitrate solution for 1 h at 56°C, rinsed by running tap water for 20 min, incubated in 0.2% gold chloride at room temperature in dark followed by distilled water rinse for 20 min and incubated in 5% thiosulphate for 5 min at room temperature and washed with distilled water and dehydrated in graded ethanol, cleared in xylene, and mounted with permount.

### Stereologic Cell Counts

Stereologic methods were employed to determine an unbiased estimate of total neurons and tyrosine hydroxylase (TH) immunopositive and Nissl-stained neurons within SNpc. Briefly, the SNpc of mice were processed for TH immunohistochemistry and Nissl counter stain on every fourth midbrain section throughout the entire extent of SNpc as described previously [67], [69]. Neurons were counted using the optical fractionator [70] using a computer-assisted image analysis system, consisting of an Axiophot photomicroscope (Carl Zeiss Vision, Hallbergmoos, Germany) comprising of a Zeiss planapochromat 100 X oil objective equipped with a computer-controlled motorized stage, a video camera, and the Stereo Investigator software (MicroBrightField, Williston, VT). Cell counts were performed throughout the entire extent of the SNpc using a standard mouse [71] atlas as anatomical reference. The total number of TH- positive neurons was calculated using the formula previously described for this method [72].

### Measurement of biogenic amines by HPLC with electrochemical detection

To determine the concentration of biogenic amines in discrete brain regions by HPLC with electrochemical detection, mice were sacrificed by decapitation and the brain was quickly removed and discrete brain regions dissected and immediately frozen and stored at -80°C. The tissue was weighed and sonicated in 0.2 ml of 0.1 M perchloric acid containing 0.01% EDTA and 25 µg/ml 3,4-dihydroxybenzylamine (DHBA) (Sigma, St. Louis, MO) as an internal standard. After centrifugation (15,000×g, 10 min, 4°C), 20 µl of the supernatant were injected onto a C-18 reverse phase Spheri 5, RP-18, 4.6 mm ×25 cm catecholamine column (BASi, West Lafayette, IN). The mobile phase consisted of 0.15 M chloroacetic acid, 0.2 mM EDTA, 0.86 mM sodium octyl sulphate, 4% acetonitrile and 2.5% tetrahydrofuran (pH = 3). The flow rate was kept at 1.5 ml/min. Biogenic amines and their metabolites were detected by an electrochemical detector, Prostar ECD Model 370 (Varian, Palo Alto, CA), with the working electrode kept at 0.6 V. Data were collected and processed on a Star Chromatography Workstation 5.52 (Varian) [67].

### Immunoblot Analysis

Immunoblot analysis for synuclein and other proteins were determined as previously described [11], [73]. For the immunoblot analysis of Akt, same procedure was used except tissue was homogenized in the TNE buffer [10 mM Tris-HCl (pH 7.4), 150 mM NaCl, 5 mM EDTA] containing protease inhibitors (5 mM PMSF, 10 µg/ml aprotinin, 10 µg/ml leupeptin, 10 µg/ml pepstatin), detergent (0.5% Nonidet P-40, 0.5% Na-Deoxycholate and 1% SDS), and a phosphatase inhibitor cocktail (Sigma).

Briefly, homogenates containing equal amount of protein were separated by SDS-PAGE, electroblotted onto a nitrocellulose membrane (BioRad, Hercules, CA), and immunoreacted with an appropriate primary antibody followed by a HRP-conjugated secondary antibodies (Kirkegaard Perry Labs Inc. MD, USA). The immunoreactive proteins were visualized by incubating the blots in the chemiluminescence substrate (Pierce, Rockford, IL) and detection with ChemiDoc XRS system (Biorad, Hercules, CA). The quantitative analyses of the immunoreactive proteins were performed with the Quantity One 1-D Analysis software (Biorad, Hercules, CA). Statistical analysis was performed using the ratios of the densitometric value of each band and its corresponding GAPDH loading control within each genotype group.

To detect various synuclein variants, following primary antibodies were used: total α-synulcein (Syn-1 mAb, BD Biosciences Pharmingen, San Jose, CA, 1∶2000); human α-synuclein (HuSyn-1 rabbit pAb, 1∶2000) [11]; β-synuclein (mAb, Research Diagnostic Inc. Concord, MA 1∶500). To detect Akt and phosphorylated Akt levels, following primary antibodies were used: total Akt (pAb), *p*Akt-Ser473 and (Cell Signalling Technology Inc. Danvers, MA) and for loading control GAPDH (Research Diagnostics Inc. Concord, MA, 1∶5000) was used.

### Statistical Analysis

Data represent mean ± SEM from groups of animals and statistical analysis applied with two-way ANOVA. When F values implied significance at a level p<0.05, Fisher's post-hoc analysis or Tukey-Kramer multiple comparison tests was applied to determine where the differences among groups arose. All statistical analyses were performed using the Prism software (GraphPad, San Diego, CA).
