# Correction: Resistance to MPTP-Neurotoxicity in α-Synuclein Knockout Mice Is Complemented by Human α-Synuclein and Associated with Increased β-Synuclein and Akt Activation

**DOI:** 10.1371/annotation/f743434e-a6bf-4c6c-a836-ae5af66035a4

**Published:** 2012-03-19

**Authors:** Bobby Thomas, Allen S. Mandir, Neva West, Ying Liu, Shaida A. Andrabi, Wanda Stirling, Valina L. Dawson, Ted M. Dawson, Michael K. Lee

There is a typographical error in the funding statement. Grant NS060885 is incorrectly published as NS06885. 

